# C1q and the classical complement cascade in geographic atrophy secondary to age-related macular degeneration

**DOI:** 10.1186/s40942-022-00431-y

**Published:** 2022-11-08

**Authors:** Ted Yednock, Donald S. Fong, Eleonora M. Lad

**Affiliations:** 1 Annexon Biosciences, 1400 Sierra Point Parkway Building C, 2nd Floor, Brisbane, CA 94005 USA; 2 Department of Ophthalmology, Duke University Medical Center, 2351 Erwin Rd, Durham, NC 27705 USA

**Keywords:** Age-related macular degeneration, Complement, geographic atrophy, C1q, Neurodegeneration

## Abstract

Geographic atrophy (GA) secondary to age-related macular degeneration (AMD) is a retinal neurodegenerative disorder. Human genetic data support the complement system as a key component of pathogenesis in AMD, which has been further supported by pre-clinical and recent clinical studies. However, the involvement of the different complement pathways (classical, lectin, alternative), and thus the optimal complement inhibition target, has yet to be fully defined. There is evidence that C1q, the initiating molecule of the classical pathway, is a key driver of complement activity in AMD. C1q is expressed locally by infiltrating phagocytic cells and C1q-activating ligands are present at disease onset and continue to accumulate with disease progression. The accumulation of C1q on photoreceptor synapses with age and disease is consistent with its role in synapse elimination and neurodegeneration that has been observed in other neurodegenerative disorders. Furthermore, genetic deletion of C1q, local pharmacologic inhibition within the eye, or genetic deletion of downstream C4 prevents photoreceptor cell damage in mouse models. Hence, targeting the classical pathway in GA could provide a more specific therapeutic approach with potential for favorable efficacy and safety.

## Background

Geographic atrophy (GA) secondary to age-related macular degeneration (AMD) is a chronic progressive neurodegenerative disorder of the retina involving the loss of photoreceptor cells (neurons) and supportive retinal pigmented epithelial cells (RPE) [1]. GA affects approximately 5 million people world-wide and is a common cause of blindness in the developed world [2]. The complement system has been implicated in GA by human genetics, retinal pathology, genomic and proteomic analyses of human tissues, and by work in animal models [3–11]. However, these studies do not address the identity of the pathway for complement activation in disease—classical, lectin, or alternative. While the alternative pathway has been implicated in numerous studies, its specific role is unclear since components of the alternative pathway are integral to the amplification of all three pathways. Further, targeting alternative pathway components has not been clinically successful [12]. Inhibition of C3 or C5 as core components of all 3 pathways has proven more promising in clinical studies, but targeted inhibition within a single pathway may provide greater safety and efficacy. In this regard, C1q, the initiating molecule of the classical complement pathway, may play an important role in GA pathogenesis. C1q and its activating substrates are present in nearly all layers of the outer retina and accumulate throughout disease progression. Further, C1q plays a unique role in synapse elimination and neurodegeneration in multiple disorders of the central nervous system [13–19] and may likewise contribute to the loss of photoreceptor cells in GA. Finally, genetic deletion of C1q or downstream C4, as well as pharmacological inhibition of C1q are protective in animal models of photoreceptor cell loss [6, 11]. Thus, C1q and the classical pathway are well positioned to be important drivers of complement activation and pathology in GA.

### Overview of the complement system

**Fig. 1:**
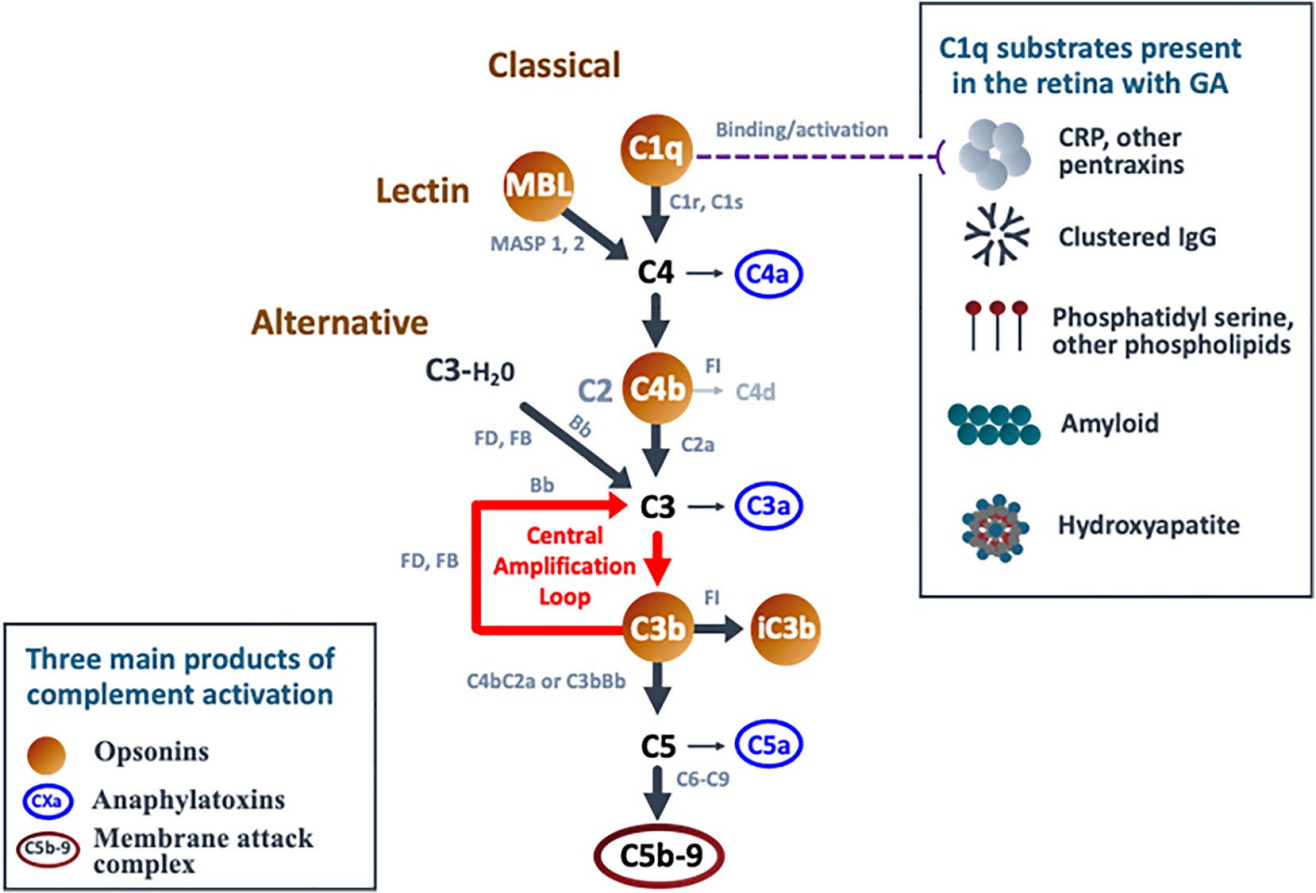
Key Components of the Complement System: C1q is the initiating molecule of the classical pathway. Binding to any of a number of substrates triggers its activation, leading to an enzymatic cascade with cleavage of the core solution-phase components C4, C2, C3, and C5 to generate activated fragments. The lectin pathway is initiated by MBL or related ficolin or collectin family members [22]. They activate the same cascade (C4, C3, and C5) as C1q by binding to a different but overlapping set of substrates, including carbohydrate and acetylated structures [21]. The alternative pathway is initiated by spontaneous solution-phase hydrolysis of C3 to generate C3b-H_2_O, which then becomes associated with the activated protease Bb to cleave other C3 molecules, generating C3b [23]. If close enough to a surface, C3b can covalently attach to initiate the surface amplification loop described above. It is important to note the distinction between the alternative complement pathway and the complement amplification loop. The alternative pathway is defined by its initiating mechanism—the spontaneous activation of C3 via non-enzymatic hydrolysis. This event leads to production of C3b, which then engages factor B and the amplification loop. However, since all three complement pathways lead to production of C3b and engage the amplification loop, this loop functions as a general “complement amplification loop” or “C3b amplification loop.” Hence, unless specifically affecting the spontaneous hydrolysis rate of C3, genetic polymorphisms or mutations that affect amplification impact the activity of all three pathways (e.g., FH, FI, FB, FD, C3, and FHR1/3). Also of note, the classical and lectin pathways will remain active as long as their substrates are present

The complement system is comprised of a core enzymatic cascade that has three major routes of activation—the classical, lectin, and alternative pathways (Fig. 1; [20]). Each pathway is defined by distinct initiating molecules that interact with different target substrates for activation, but all produce 3 main outcomes: target opsonization, release of anaphylatoxins, and target membrane damage (Fig. 1). Opsonization refers to coating the target surface with complement activation products, or opsonins (e.g., C1q, C4b, C3b, iC3b), that are recognized by phagocytic immune cells. Anaphylatoxins are soluble activation products (C4a, C3a, C5a) that increase vascular permeability, regulate local cell activity, and recruit immune cells. The membrane attack complex is comprised of complement components (C5b, C6-C9) that form a pore structure resulting in membrane dysfunction or cell lysis. Due to a high degree of pathway regulation, upstream activation can lead to varying levels of immune cell recruitment and opsonization without resulting in downstream lysis.

C1q is the initiating molecule of the classical pathway, while mannose-binding lectin and the highly related molecules collectin-10 and -11 and ficolin-1, -2, and -3 (together referred to as “MBL” in this review) are the initiating molecules of the lectin pathway [21, 22]. They function as pattern recognition molecules that bind to a broad spectrum of substrates found on pathogens, damaged tissue, and tissue debris (Fig. 1). They are designed to focus robust complement activation on a select set of targets. In contrast, the alternative pathway is stochastic, with spontaneous solution-phase hydrolysis of complement component C3 that leads to random tagging of nearby surfaces with C3b for pathway activation [23]. The alternative pathway functions to detect pathogens or substrates that may not otherwise be recognized by more selective immune receptors. While diffuse activation of the alternative pathway is ongoing throughout the body, classical and lectin pathway activation is initiated by specific substrates and ceases when the substrates are cleared. However, if activating substrates are persistent (produced faster than can be removed or resisting removal) ongoing complement activation can occur, resulting in significant tissue damage. In GA, numerous C1q-activating substrates are present at the earliest stages of disease and continue to accumulate with disease progression as components of drusen and drusenoid deposits, as well as normal and stress-related components of cell membranes. As shown in Fig. 1, these substrates include pentraxins such as C-reactive protein (CRP) and serum amyloid protein (SAP), phospholipids such as phosphatidyl serine and lysophospholipids, hydroxyapatite, immunoglobulins, and amyloid material (discussed further below; [22, 24–29]). Hence, ongoing classical complement activation is a likely component of the chronic inflammatory response in GA.

It is important to note that all 3 complement pathways lead to the cleavage of C3 to form C3b as a central component of the complement system. C3b can engage the protease factor B to cleave additional C3 and generate more C3b. This central amplification loop functions to amplify the amount of C3b bound to the substrate surface and is an integral part of all 3 complement pathways, thereby enhancing their impact [22, 30]. Since the amplification loop involves factors B and D, it is often confused with the alternative complement pathway, which is initiated by spontaneous, solution-phase hydrolysis of C3 (C3-H2O) and depends upon factors B and D for amplification. However, the amplification loop is a component of all three pathways, and genetic mutations that affect the amplification loop (including C3, complement factor H-related (FHR) 1/3, and factors B, D, I, and H) affect the activity of all three pathways. Greater detail of these pathways is provided in the legend to Fig. 1 and in the text below.

### Human genetics implicate the complement system in GA

Human genetics strongly implicate the involvement of the complement system in GA. Variants in at least 6 complement-related genes are associated with disease, including CFH, CFHR1/3, C2/CFB, C3, CFI, C9 [3, 4, 32–35]. Together, variants in complement-related genes account for more than half of AMD genetic risk [3, 36], and the altered protein products of these genes have been directly linked to regulation of complement activity (Table 1). Other genetic loci are strongly and independently linked to GA, but can work in conjunction with complement variants to augment GA progression. The ARM2/HTRA1 locus, in particular, has been associated with increased neovascular AMD [37–40].

**Table 1:** Genes associated with GA/AMD

Gene	Protein	Activity	Impact of mutation/polymorphism on protein product function	Mutation impact on complement activity	GA risk
CFH	Factor H (FH)	Cofactor for FI-mediated cleavage of C3b; Competes C1q binding; Competes FHR1	Decrease surface binding of FH decreases FI cleavage; allows C1q binding	Increase	Increase OR = 2.7–7.4
CFHR1/3	FHR1 and / or FHR3	Stabilizers C3b; enhances C1q binding; Competes FH	Gentic deletion allows more FH binding, greater FI cleavage of C3b, less C1q binding	Decrease	Decrease OR = 0.35
CFI	Factor I (FI)	Cleaves C3b to inhibit cascade activity and to produce Ic3b for opsonization/phagocytosis	Reduced FI levels / activity	Increase	Increase OR up to 22
C2	C2	Drives classical / lectin activation of C3	Uncharacterized	Increase	Decrease OR = 0.47–0.49
CFB	Factor B (FB)	Drives alternative / AP amplification via activation of C3	Decrease activity	Decrease	Decrease OR = 0.41–0.54
C3	C3	Activation leads to C3b as opsonin and plateform for AP amplification or C5 cleavage	Resistance to inactivation by FI cleavage	Increase	Increase OR 2.8
C9	C9	Component of membrane attack complex(MAC)	Increased MAC polymerization	Increase	Increase OR 2.2

Reference for Table 1: CFH: 23, 37, 38, 41–44; CFHR1/3: 32, 39, 45–48; FI: 35, 49, 50, 54; C2/CFB: 51, 52, 53, 55-57; C3: 35; C9: 58

The most compelling aspect of the genetic evidence is that variants known to increase complement activity are associated with increased risk of AMD, while those that decrease complement activity provide protection. For example, a common variant of CFH (Y402H) that generally leads to enhanced complement activity is associated with a 3- to sevenfold increased risk of AMD [41, 42], and a common genetic deletion of FHR1 that decreases complement activity reduces risk of AMD by 60% [32, 43, 44]. The impact of genetic polymorphisms in AMD is further discussed in Table 1.

While human genetics point to increased complement activity in GA, they do not specifically address how complement is being activated in disease. The alternative pathway has been historically implicated in AMD pathobiology because many of the affected genes impact the stability of C3b, the initiating component of the amplification loop. However, as discussed above, C3b and its amplification loop are central components of all 3 complement pathways, with C3b functioning as a key opsonin and a critical element of both C5 convertases (i.e., C3b working in conjunction with C4b2a or C3bBb for C5 convertase activity; Fig. 1).

### Complement in GA pathology

**Fig. 2:**
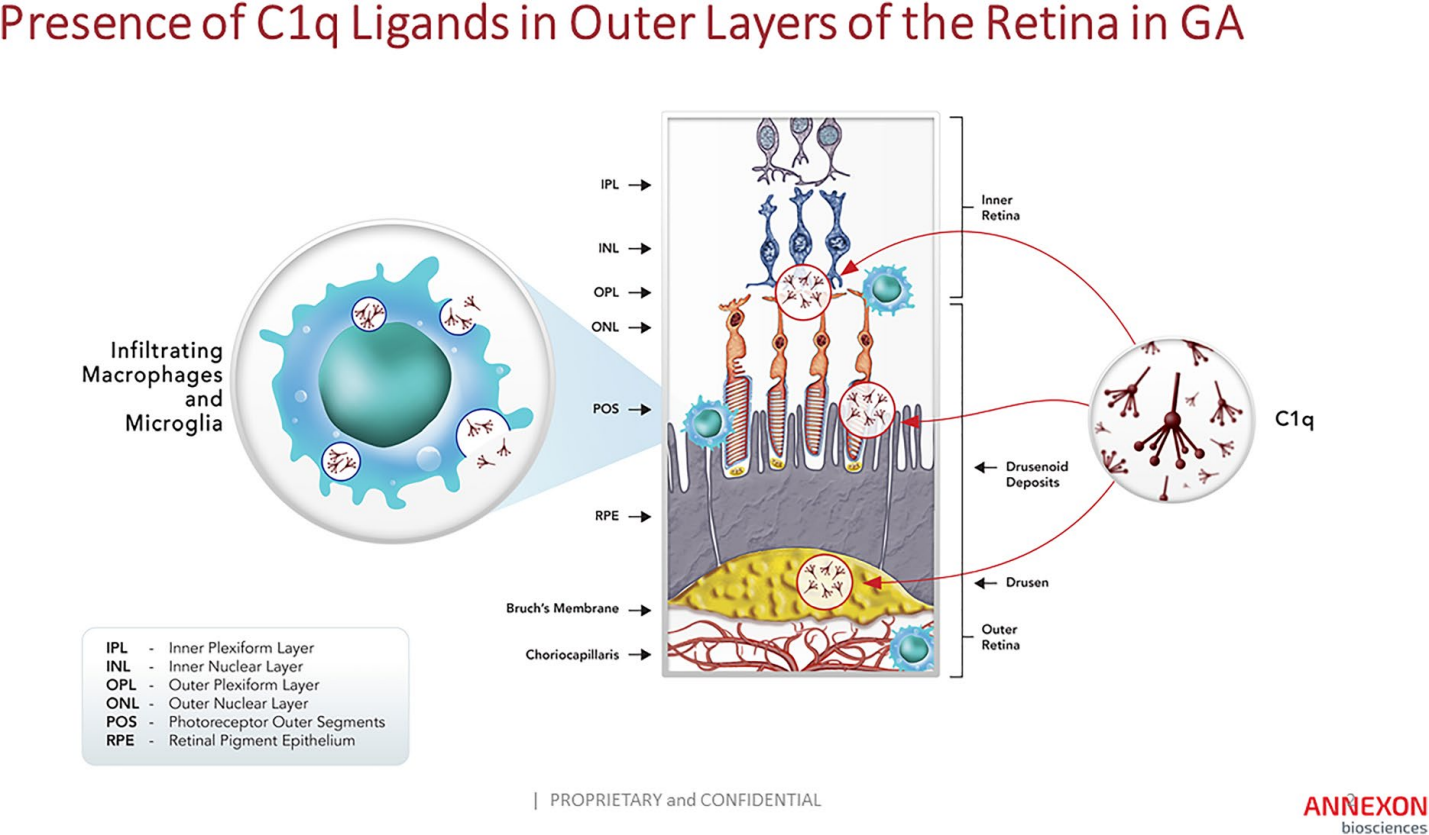
Expression and Deposition of C1q in Outer Layers of the Retina in GA: C1q is deposited in multiple locations within the outer retina, including on photoreceptor cell synapses within the OPL, on photoreceptor cell outer segments below the ONL, and on drusen within the RPE and BM. C1q is expressed locally within these layers by infiltrating microglial cells and / or macrophages

GA affects the outer most layers of the retina—the photoreceptor cells, RPE cells, and vascular choroid layer—and involves the presence or abnormal accumulation of complement-activating substrates within these layers ([4, 5, 36]; Fig. 2). The unique oxidative and metabolic environment of the outer retina leads to damage of light-collecting membranes, the photoreceptor cell outer segments (POS), that undergo a process of distal shedding and proximal renewal [59]. Shed POS exhibit phosphatidyl serine on their outer membrane, facilitating their recognition by RPE for uptake and removal. However, in AMD, damaged membranes and other lipid-rich cellular components accumulate as drusen and drusenoid deposits below and above the RPE [36, 60, 61]. These deposits contain a number of complement-activating substrates that are specifically recognized by C1q, including CRP, SAP, phosphatidyl serine, hydroxyapatite, lysophospholipids, immunoglobulins, and amyloid material [8, 22, 24–29, 62, 63]. A compromise in the blood-retinal barrier due to RPE dysfunction allows leakage of serum proteins, including C1q and other complement components, into the retina from the underlying choriocapillaris [6]. C1q, either alone or in conjunction with CRP and other pentraxins, binds to phosphatidyl serine exposed on POS and on damaged RPE membranes [6], as well as to the numerous substrates present in drusen. Local complement activation leads to recruitment of macrophages and microglia, which produce additional C1q and other complement components. POS normally lack expression of membrane-bound complement regulatory proteins (such as CD46, CD55; [6]), while expression of these proteins is down-regulated on the surface of damaged RPE, making membranes within both retinal layers exposed to complement attack [64]. With ineffective clearance and continued accumulation of drusen and drusenoid deposits, C1q binding and classical complement activation will continue, along with engagement of the downstream amplification loop. Therefore, the unique structure and function of the outer retina—with highly metabolic, membrane-rich cells that are exposed to light in an oxygen-rich environment—makes it particularly vulnerable to dysregulation of the complement system.

### Animal models of GA

To study the role of complement in RPE and photoreceptor cell damage, numerous animal models have been developed through direct damage to these cell types, including exposure to blue light to damage RPE, white light to induce photoreceptor cell damage, NaIO_3_ administration to selectively kill RPE, focal laser damage to disrupt the blood/retinal barrier, and immunization against photo-oxidative metabolites to cause direct immune attack [6, 65, 66]. These studies consistently show increased retinal expression of complement components within the classical, amplification loop, and lytic pathways (RNA and protein; [6, 10, 67, 68]). Results suggest an important role for both the classical and alternative/amplification pathways, with dominance by the alternative/amplification pathway, in many models [6, 68–70]. Again, it is impossible to distinguish between the alternative pathway and the central amplification loop in these studies because components identified in this work, such as C3 and factors B, D, H, and I, are common to both.

While these results are often cited as support for the alternative pathway as a key driver of GA, it is important to note that many studies focus on acute induction of disease and tissue damage over a period of days to weeks rather than chronic disease in which ongoing accumulation of specific complement substrates may induce persistent complement activation. It is possible that amplification loop plays an outsized role in disease initiation by enhancing a complement signal generated through early accumulation of damaged cells or specific complement substrates. Further, the activation or regulation of the classical pathway in mice is distinct from that in humans and other species, with the classical cascade generally being highly restrained in mice [7, 71–74]. Consistent with this idea, exposure of POS to human serum in vitro leads to C3 deposition on their surface that is driven in large extent by the classical pathway, while C3 deposition upon exposure to mouse serum is entirely dependent on the alternative pathway [6].

### Clinical trials of complement inhibitors

The “alternative pathway hypothesis” was rigorously tested in AMD in 2 large phase 3 studies of lampalizumab, a potent factor D (FD) inhibitor dosed via intravitreal administration [12]. FD inhibition would block alternative pathway initiation as well as the amplification loop (Fig. 1). Patients received intravitreal lampalizumab (10 mg/eye/month) or sham injections for 48 weeks, tracking GA lesion size and visual function. Unfortunately, both phase 3 studies were negative, even in a subset of patients that carried genetic variants of complement factor I associated with AMD risk [12]. A separate and smaller study done with another inhibitor of the alternative pathway was also negative (CLG-561, anti-properdin Fab; NCT02515942). There are many potential explanations for these results, including a need to capture patients before advanced disease ensues, for systemic rather than local inhibition, for different/more potent drug molecules that better penetrate the retina, or the need to inhibit additional or different complement pathways upstream of the amplification loop. Likewise, targeting downstream C5—either with intravitreal (LFG316, NCT01527500; [75]) or systemic (eculizumab; NCT00935883; [76]) administration—or by targeting C5 in combination with the alternative pathway (LFG316 + CLG561; NCT02515942) were also not successful in early phase clinical studies, potentially suggesting that the alternative or common lytic pathways are not key drivers of pathology in AMD; however, these latter studies were either small (n < 40/arm; LFG316/CLG561) or of limited duration (6 months; eculizumab).

Inhibition of C3, the central component of all three complement pathways, has been evaluated in one phase 2 [78] and two phase 3 studies with pegcetacoplan, an anti-C3 peptide conjugated to 40 kD polyethylene glycol (PEG). At 24 months, compared to sham, monthly and every-other-month treatment with intravitreal pegcetacoplan significantly reduced GA lesion growth by 22% (p = 0.0001) and 18% (p = 0.0002), respectively in the OAKS studyand by 19%% (p = 0.00004) and 16% (p = 0.003), respectively, in the DERBY study (company press release).

In GATHER1, a trial investigating the efficacy of C5 inhibition with a different compound (avacincaptad pegol; NCT02686658), there was a statistically significant 27.4% (p = 0.0072) reduction in GA lesion development at 12 months [77]. The second trial, GATHER2 showed a 14.3% (p = 0.0064) reduction in mean rate of growth at 12 months (company press release).

Treatment with either drug, however, resulted in an increase in development of exudative AMD/neovascularization in the treated eye (4–6% with C3 inhibition vs. 2% sham, and 10% with C5 inhibition vs. 3% sham). A potential explanation may be that, although C3 and C5 inhibition prevent neovascularization in several acute models used for GA, it can promote neovascularization in other models of retinal damage [79] or can prevent tissue repair [80–84]. Also, C3-mediated phagocytosis has been shown to be protective of RPE in a murine model of retinal damage [85]. Alternatively, the PEG carrier molecule common to both drugs may itself induce complement activation and vascular growth, as shown with direct subretinal injection [86]. However, in this model, PEG injections were administered in the subretinal space and induced direct tissue damage, unlike the scenario in clinical studies which employed intravitreal PEGylated drugs. Overall, the results of these clinical trials are encouraging, indicating that intravitreal administration of inhibitors of C3 or C5 can be effective, each providing close to 30% reduction in lesion growth at 1 year. Nevertheless, since these approaches fully inhibit the downstream components of all three complement pathways, they do not address the questions posed by human genetics: how C3 is being activated in AMD and how best to modulate it for therapeutic benefit.

### Potential role of the classical complement pathway in GA

Multiple lines of evidence suggest that the classical pathway may be a key initiator of complement activation in GA. First, C1q is present in all layers of the outer retina in GA along with numerous C1q-activating ligands that accumulate throughout disease initiation and progression. Second, C1q and the classical pathway are implicated by retinal pathology in GA and in animal models of GA. Third, in RPE culture models, C1q triggers C5b-9 deposition on basement membrane components and contributes to induction of the inflammasome and cell necrosis. Fourth, inhibition of C1q and downstream C4 is protective in animal models of photoreceptor cell loss. Fifth, C1q accumulates on photoreceptor cell synapses in mice with age and light-induced injury, consistent with the unique role it plays in synapse elimination and neurodegeneration in other disorders. Each of these points is discussed in greater detail below.

#### C1q and C1q-activating ligands are present in the retina with GA

**Fig. 3:**
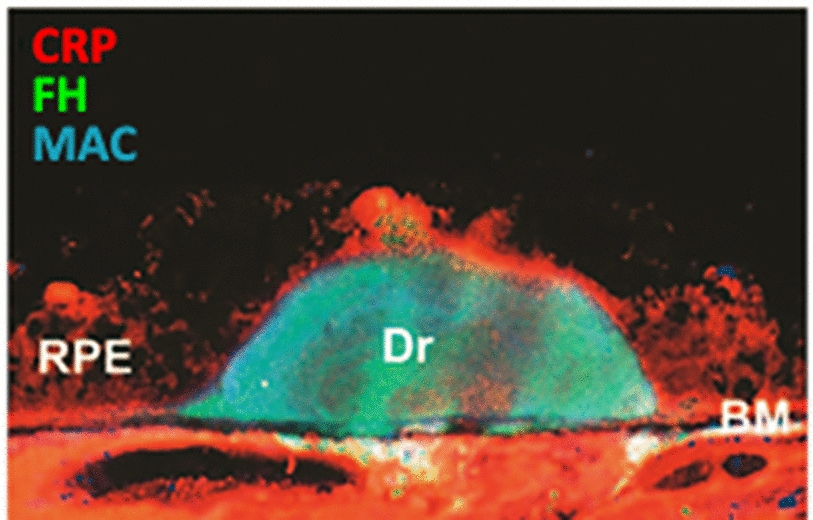
CRP Associates with drusen and RPE in GA: Confocal image of the retina of a patient with AMD showing drusen (Dr) stained for CRP (red) and factor H (green) [88]

C1q is highly expressed in infiltrating macrophages within all layers of the outer retina (OPL, POS, RPE, and choroid layers) in GA (Fig. 2; [6, 11, 87]). In addition, disruption of the RPE cells and their ability to maintain a tight blood-retina barrier allows increased influx of C1q from the circulation along with other blood proteins [6]. Activating ligands for C1q in GA include surface bound CRP, SAP, membrane-exposed phosphatidyl serine, hydroxyapatite, lysophospholipids, immunoglobulins, and amyloid material [8, 22, 24–29, 62, 63]. Many of these ligands are associated with RPE and Bruch’s membrane or are components of drusen and drusenoid deposits (Figs. 1, 2, 3; [88–90]). C1q activation on CRP is increased by FHR1, a soluble complement regulatory protein that has been genetically linked to AMD. FHR1 enhances C1q activation and stabilizes downstream C3b (Table 1; [51]). Decreased expression of FHR1 reduces complement activation potential and is protective against AMD. Further, C1q activation may be enhanced by autoantibodies associated with disease [91–93]. In mice, generation of antibodies against oxidation products unique to the outer retina leads to classical complement activation, with downstream C3 deposition in Bruch’s membrane, macrophage infiltration and RPE damage [50, 66]. In addition, the presence of these oxidation products with C1q in drusen activates the NLPR3 inflammasome in cultured RPE, contributing to RPE necrosis [94]. Interestingly, genetic deletion of C1q prevented inflammasome activation in a mouse model of photoreceptor cell damage [67].

#### C1q/classical pathway implicated in GA by human and animal pathology

**Fig. 4:**
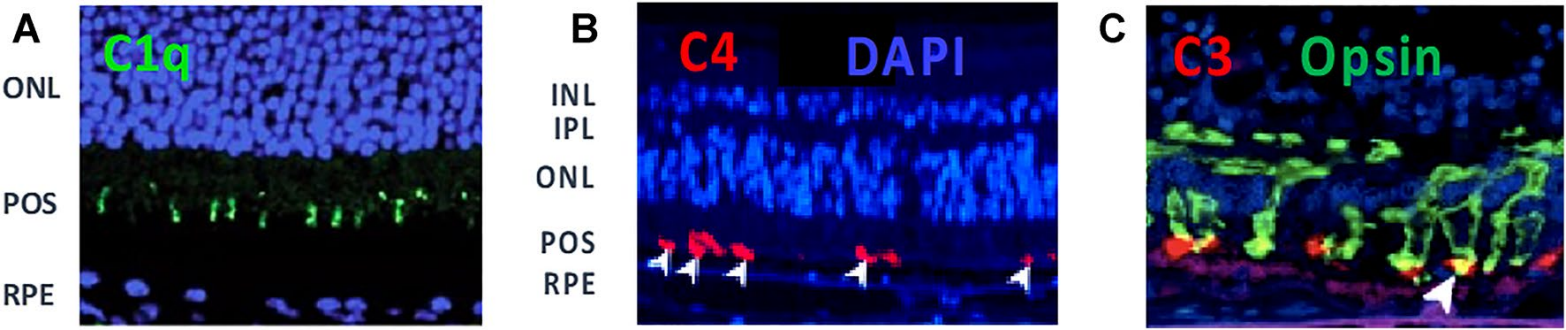
Complement Deposition on POS in Mouse Models of GA and in GA Patient Tissue: Panel **A**: Image of mouse retinal Sect. 5 h after RPE damage caused by intravenous injection of NaIO_3._ Immunofluorescence shows C1q deposition on POS, which occurs in conjunction with C3 prior to decrease in retinal function by day 7; Panels **B** and **C**: Images of retinal sections from patients with GA. Immunofluorescence shows C4 and C3 deposition on photoreceptor cell outer segments (POS) in a healthy region of retina just outside the edge of an expanding lesion, consistent with complement activation preceding loss of photoreceptor neurons. C4 deposition directly implicates the classical and/or lectin pathways since it occurs upstream of C3 and the alternative or amplification pathways [6]

POS exhibit the C1q-activating ligand phosphatidyl serine on their surface as part of the daily light cycle [95]. This ligand is normally recognized by soluble proteins that bridge POS, as they are shed from photoreceptors, to integrin receptors on RPE for phagocytosis. However, aberrant exposure to C1q, either from serum proteins across the blood-retinal barrier or through local production, may help drive complement activation in GA lesion development or expansion. Following retinal damage in mice, POS rapidly become coated by C1q and downstream complement activation products C4b and C3b (Fig. 4; [6]). The POS are then phagocytosed by infiltrating macrophages, and photoreceptor cells are lost. Genetic deletion of C4 as part of the classical/lectin pathway and inhibition of FD within the alternative/amplification pathway provided full protection against photoreceptor cell loss [52–58]. When isolated POS are exposed to human serum in vitro, they activate C4 deposition via the classical pathway and C3 deposition by both the classical and alternative pathways to facilitate phagocytosis by macrophages. There is no apparent involvement of the lectin pathway in this in vitro system [78]. In tissue from patients with GA, C4 and C3 are deposited on POS of both rods and cones in areas of healthy retina just outside the expanding edge of lesions, supporting the idea that C1q recognition of POS is contributing to GA lesion development or expansion (Fig. 4; [6]).

#### C1q triggers C5b-9 deposition and contributes to induction of the inflammasome in cultures of RPE

**Fig. 5:**
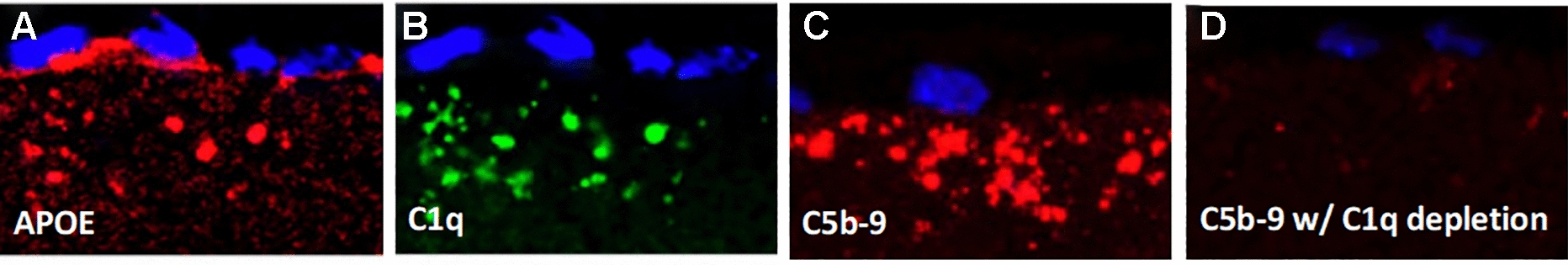
ApoE-rich Deposits That Form Below Cultured RPE Cells Activate the Classical Complement Pathway:Primary human RPE cells grown on porous supports produce globular extracellular deposits. Cultures were exposed to normal or complement-depleted human serum and then processed for immunohistochemistry with various antibody markers. **A**. Anti-ApoE with no serum exposure; **B**. Anti-C1q after exposure to normal human serum; **C**. Anti-C5b-9 after exposure to normal human serum; **D**. Anti-C5b-9 after expsoure to C1q-depleted human serum. Depletion of factor B had no impact on C5b-9 staining, implicating C1q-dependent/classical cascade activation [96]

In vitro culture of RPE cells results in deposition of apolipoprotein E (ApoE)-rich drusen-like material that binds and activates C1q, leading to complement activation and deposition of C5b-9 [96]. Complement activation was dependent on the classical, but not the alternative pathway, as determined by serum depletion of either C1q or factor B (Fig. 5). In a separate study, in vitro treatment of lipoparticles (containing ApoE) with hepatic lipase led to production of lysophospholipids that bound C1q and induced complement activation. Again, this activity was dependent on the classical, but not the alternative pathway, and C1q activation was facilitated by CRP [28]. Lysophospholipids are present at high levels in drusen, while genetic variants of ApoE and lipase C, hepatic type (LIPC) gene are both linked to GA. Polymorphisms that result in reduced levels of hepatic lipase are associated with GA protection. In a separate in vitro model, C1q worked in conjunction with other drusen components to induce the inflammasome in RPE cells, leading to RPE necrosis [94].

#### C1q/classical cascade inhibition is protective in animal models of disease

**Fig. 6:**
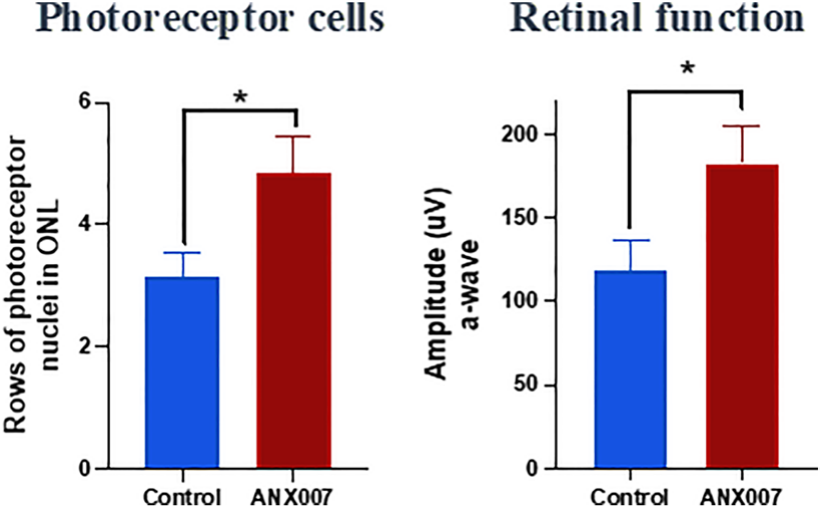
C1q Inhibition Protects Photoreceptor Cells and Retinal Function: Anti-C1q was given via intravitreal administration 7 days after initiation of photo-oxidative damage. Measures were taken one week after dosing. Amplitude measures for a wave (left graph) and b wave (not shown) were taken with flash intensity of 1.9 log cd.s/m^2^ [11]

**Fig. 7:**
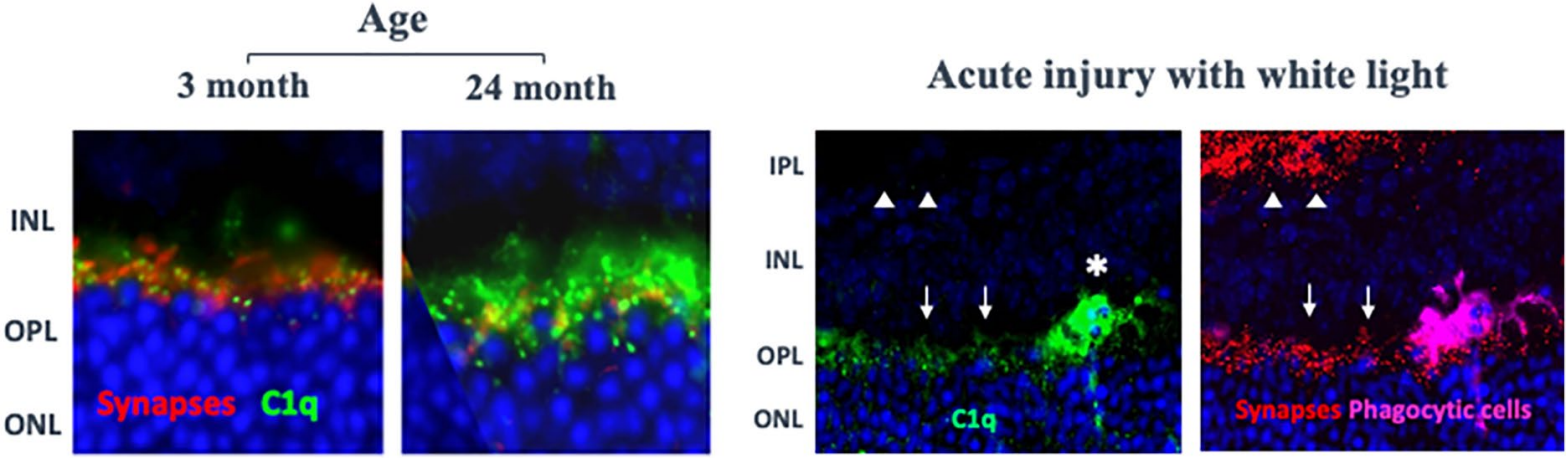
C1q Binds Photoreceptor Cell Synapses With Age or Retinal: Left images: Mice were aged from 3 to 24 months. Retinal sections were stained with anti-synaptophysin and anti-C1q (antibody 4.8, Abcam); Annexon, data on file. Right images: Balb/c mice were exposed to white light (28-29 k lux) for 4 h, and after an additional 20 h, retinal sections were prepared and stained with anti-C1q as above. Note C1q staining of both synapses (arrows) and infiltrating cells (asterisk); adjacent retinal section stained with bassoon (presynaptic marker: red) and IBA-1 (infiltrating macrophages or microglia: magenta). Note C1q overlap with synapses of the photoreceptor cells (arrows) in the OPL but not synapses within the IPL (arrow heads) [108]. Abbreviations, see figure.

In a model of photoreceptor cell damage involving prolonged exposure to white light, macrophages infiltrate the outer retina where they express high levels of C1q. Genetic deletion or pharmacological inhibition of C1q provided protection against photoreceptor cell loss and preserved receptor cell function, even when inhibition was provided after damage had occurred (Fig. 6; [11] also see Fig. 7 for expression of C1q in a similar model). Interestingly, while local (intravitreal) administration of an anti-C1q antibody was protective, systemic administration was not—presumably due to local production of C1q in the retina and to lower levels of antibody penetration into the eye with systemic dosing. Further support is provided by 2 additional animal models, where genetic deletion of C4 was protective against photoreceptor cell loss following RPE damage induced by NaIO_3_ or by direct laser damage [6].

#### C1q’s unique role in synapse elimination and neurodegeneration

Photoreceptor cell stress in GA caused by decline in RPE function and other factors may lead to a process of synapse elimination, neuroinflammation and neuronal loss that is common in multiple neurodegenerative disorders. In development, C1q recognizes the surface of weaker supernumerary synapses, activating the classical complement pathway and recruiting microglial cells to prune the opsonized synapses away from neurons [13, 97]. The role of C1q in sculpting neuronal circuitry was first shown in the visual pathway examining synaptic connections in the lateral geniculate and has more recently been shown to occur during development with synapses in the outer retina [98]. After development, C1q accumulates on synapses with normal aging, increasing up to 300-fold in aged mice relative to younger animals [15]. Age-related accumulation may reflect changes on the synapse surface that resemble those in development and appears to place synapses in the adult at risk for aberrant removal. In GA, with declining RPE function and neuronal stress, C1q on synapses may activate the classical pathway, and as in other neurodegenerative disorders, result in compromised synapse function and microglial cell recruitment [99]. Microglial cells then prune complement-coated synapses away from neurons, depriving neurons of trophic support, and cause neuroinflammation that adds to neuronal damage and loss. C1q inhibition has been shown to be protective in multiple animal models of neurodegeneration by blocking complement-mediated synapse elimination in the central nervous system (CNS), including models of Alzheimer’s disease [16, 19, 100], spinal muscular atrophy [101], glaucoma [14, 18], photoreceptor cell damage [11], frontotemporal dementia [17], traumatic brain injury [102, 103], and Huntington’s disease [104]. The fact that C1q is involved in synapse pruning in the outer retina during development [98] and that genetic deletion or pharmacological inhibition of C1q is protective against synapse loss and neuronal damage in several models of glaucoma demonstrates that this mechanism is relevant to the retina [14, 18]. Consistent with a potential role in GA, we have found that C1q accumulates in the OPL on photoreceptor cell synapses in mice with both age and acute light damage. C1q is also strongly expressed by phagocytic cells that infiltrate the OPL following light damage (Fig. 7) and in other animal models of photoreceptor cell loss [6, 11]. Thus, declining RPE function and photoreceptor cells stress may augment the decline of retinal function and integrity through a previously-described process of complement-mediated neurodegeneration [45–49].

### Advantages of targeting C1q in GA

**Fig. 8:**
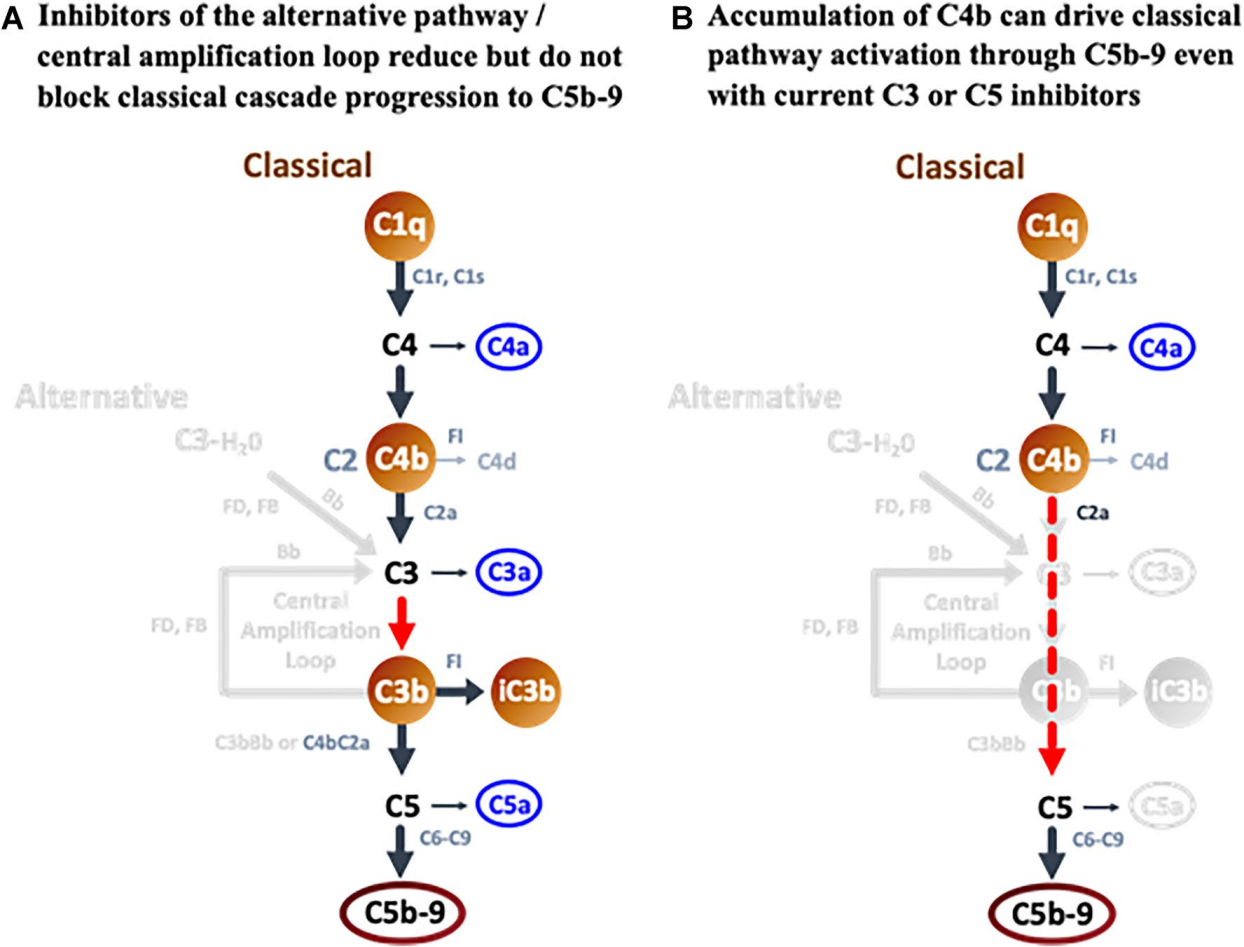
Activation of C5 in the Presence of C3, C5, or Alternative Pathway Inhibitors

The initial success of clinical trials with C3 and C5 inhibitors in GA extends insights from human genetics, indicating a role for complement in GA progression beyond disease initiation. Further, it suggests that efficacy can be achieved in the outer retina through intravitreal administration of a complement inhibitor. However, C3 and C5 inhibitors block the central and terminal aspects of all three complement pathways. Animal studies have shown that these approaches can negatively impact retinal health and facilitate neovascularization following retinal damage. While upstream targeting of the alternative pathway has not been successful to date, targeting upstream components of the classical pathway, in particular C1q, may provide a therapeutic advantage. In chronic diseases such as GA where classical cascade substrates accumulate and persist, there will be continuous opportunity for C1q binding and classical complement activation, with C4b, C3b, and C5b-9 production (Fig. 1). Following alternative pathway inhibition, C3b levels would be reduced [105], but there would still be activation of C3 and C5 through the C4b2a convertase, particularly in a chronic disease setting (Fig. 8A). Remarkably, even with C3 or C5 inhibition, chronic or excessive accumulation of C4b can drive C5b-9 activation directly (Fig. 8B; [31]). In contrast, preventing the focused binding of C1q to its substrates would block all downstream classical cascade components and their amplification to reduce overall complement activation, while leaving the clearance and tissue repair functions of the lectin and alternative complement pathways in place. Given supporting evidence that C1q is produced locally in the retina, that activating substrates for C1q are present in all layers of the outer retina in GA, and that C1q plays a unique role in synapse elimination and neuronal damage in multiple neurodegenerative disorders, the classical pathway is well positioned for therapeutic intervention in GA. In a recent phase 1 clinical study, intravitreal administration of an inhibitory antibody against C1q was able to fully reduce free C1q levels for at least one month, as measured in aqueous humor [106]. These findings were supported by preclinical studies in nonhuman primates, which additionally showed penetration of the drug into the outer layers of the retina with elimination of free C1q in the retina, optic nerve head and choroid [107]. Hence, the rationale for targeting the classical pathway as a mediator of tissue damage in GA is currently being tested in phase 2/3 studies.

## Summary

There are strong human genetic data demonstrating a role for complement in AMD pathobiology that is further supported by in vitro and in vivo model studies, and, importantly, by clinical data. These studies indicate that the complement system is a key component of pathology, but they do not address how complement is being activated in AMD—via classical, lectin, or alternative pathways. Clinical studies focused on the alternative pathway have been unsuccessful to date, while studies targeting downstream activity of all complement pathways (C3 and C5) have shown promise. C1q is well positioned to be a key driver of complement activity in AMD, with local expression by infiltrating phagocytic cells and numerous C1q–activating ligands that are present at disease initiation and accumulate with AMD progression. Genetic deletion of C1q, local pharmacologic inhibition within the eye, or genetic deletion of downstream C4 provides protection in several mouse models of photoreceptor cell damage. C1q’s presence on photoreceptor synapses with age and damage is consistent with its role in synapse elimination and neurodegeneration in many other CNS disorders. While blocking downstream components of all three pathways has provided promise in clinical studies, targeting the classical pathway in GA would provide a more selective approach with the potential for increased efficacy and safety.

## Data Availability

Not applicable. The paper is a review paper.

## Abbreviations

AMD: Age-related macular degeneration
ApoE: Apolipoprotein E
C3-H2O: Hydrolysis of C3
CNS: Centra nervous system
CRP: C-reactive protein
FD: Factor D
GA: Geographic atrophy
LIPC: Lipase C, hepatic type
MBL: Mannose-binding lectin and the highly related molecules collectin-10 and -11 and ficolin-1, -2, and -3
OPL: Outer plexiform layer
PEG: Polyethylene glycol
POS: Photoreceptor cell outer segments
RPE: Retinal pigmented epithelial cells
SAP: Serum amyloid protein
